# ZK53 enhances tumor cell susceptibility to ferroptosis via ClpP-mediated mitochondrial dysfunction

**DOI:** 10.3389/fonc.2025.1594840

**Published:** 2025-11-21

**Authors:** Songjun Dai, Tingting Zhang, Xiaoyan Dai, Keke Zheng, Hong Qian, Zhou Tong, Qiang Zhang

**Affiliations:** 1 Department of Biophysics, Second Affiliated Hospital, Zhejiang University School of Medicine, Hangzhou, Zhejiang, China; 2 Zhejiang Provincial Key Laboratory of Silkworm Bioreactor and Biomedicine, College of Life Sciences and Medicine, Zhejiang Sci-Tech University, Hangzhou, Zhejiang, China; 3 Department of Medical Oncology, The First Affiliated Hospital, Zhejiang University School of Medicine, Hangzhou, China

**Keywords:** ferroptosis, drug screening, ZK53, CLPP, mitochondrial dysfunction, cancer therapy

## Abstract

Ferroptosis is an iron-dependent form of regulated cell death driven by lipid peroxidation. This process has garnered increasing attention due to its potential as a promising therapeutic strategy for cancer. In this study, through functional screening, we identified ZK53, a small molecule that sensitizes cells to the ferroptosis inducer RSL3. Comprehensive functional characterization confirmed that ZK53 effectively enhances ferroptosis across multiple cell lines. Mechanistically, both knockdown and overexpression experiments demonstrated that ClpP, the target of ZK53, is a previously unrecognized pro-ferroptosis factor. Notably, ZK53 enhances ferroptosis via ClpP dependency and promotes cell death by inducing mitochondrial dysfunction. *In vivo*, ZK53 synergized with ferroptosis inducers IKE to significantly inhibit tumor growth in xenograft models by promoting ferroptosis. Taken together, our findings identify ClpP as a novel target for ferroptosis and suggest that ZK53 may serve as a promising candidate for enhancing ferroptosis-based cancer therapy.

## Introduction

Ferroptosis is an iron-dependent form of regulated cell death, driven by excessive lipid peroxidation (1). Central to its regulation is the enzyme glutathione peroxidase 4 (GPX4), which detoxifies phospholipid peroxides in a glutathione (GSH)-dependent manner, thereby maintaining redox homeostasis (2). When GPX4 activity is impaired, polyunsaturated fatty acid (PUFA)-containing phospholipids (PLs) become highly susceptible to peroxidation, a process further amplified by acyl-CoA synthetase long-chain family member 4 (ACSL4) and lipoxygenases (ALOX) (3–5). The resulting build-up of lipid peroxides compromises membrane integrity, ultimately triggering ferroptotic cell death (6).

Ferroptosis plays a crucial role in cancer biology as an intrinsic tumor-suppressive mechanism, regulated by key tumor suppressors such as TP53, BAP1, KEAP1, and MLL4 (7, 8). These tumor suppressors promote ferroptosis by modulating redox homeostasis, lipid metabolism, and iron regulation, thereby constraining tumor initiation and progression (9–13). Meanwhile, certain cancer cells undergo metabolic reprogramming, experience heightened oxidative stress, and acquire specific genetic alterations that increase their susceptibility to ferroptosis (14–16). This inherent vulnerability makes ferroptosis a critical determinant of cancer cell survival, where disrupting ferroptotic defense mechanisms can selectively eliminate malignant cells while sparing normal tissues (17). This selective elimination underscores ferroptosis as a promising targeted therapeutic strategy in cancer treatment.

Despite its tumor-suppressive role, many cancer cells develop resistance to ferroptosis through a variety of evasion mechanisms. These mechanisms enable the cells to survive oxidative stress and lipid peroxidation (18–22), ultimately compromising the effectiveness of ferroptosis as a therapeutic strategy. To overcome this challenge, targeting ferroptosis resistance pathways offers a promising approach to restore sensitivity to ferroptosis in resistant cancer cells and tumors (23, 24). One potential strategy involves modulating the expression or activity of oncogenic or tumor-suppressive proteins that regulate ferroptosis resistance. By targeting these regulatory proteins, it may be possible to circumvent ferroptosis-associated drug resistance and, in turn, enhance the efficacy of cancer therapies (25, 26).

Among the emerging regulators of ferroptosis, mitochondria play a particularly critical role (27–29). Mitochondrial proteins such as dihydroorotate dehydrogenase (DHODH), optic atrophy 1 (OPA1), and components of the electron transport chain (ETC), including complex I, have been identified as key modulators of ferroptosis sensitivity (17, 30, 31). Their involvement suggests a strong link between mitochondrial function and ferroptotic cell death, positioning mitochondria as a promising target for therapeutic intervention (32–34). Pharmacological modulation of mitochondrial ferroptosis regulators, particularly in combination with ferroptosis inducers, offers a compelling strategy to overcome resistance and enhance therapeutic efficacy in refractory cancers (17, 35).

ClpP, a highly conserved serine protease located in the mitochondrial matrix, is a central component of the mitochondrial protein quality control machinery (36). By forming a proteolytic complex with the ATP-dependent unfoldase ClpX, ClpP selectively degrades misfolded or damaged proteins to maintain mitochondrial proteostasis and bioenergetic function (37). Beyond this canonical proteolytic role, ClpP regulates mitochondrial stress responses, metabolic adaptation, and apoptosis (38). Several mitochondrial enzymes, including those involved in the tricarboxylic acid (TCA) cycle and respiratory complexes, have been identified as molecular targets of ClpP, underscoring its broad influence on mitochondrial metabolism (38, 39). Given these multifaceted physiological roles, ClpP has emerged as a potential therapeutic target, particularly in cancer, where its dysregulation can induce mitochondrial dysfunction and cell death (38, 39). However, its potential involvement in ferroptosis remains largely unexplored.

In this study, we conducted a mitochondrial-targeted drug screen and identified ZK53, a small molecule that significantly increases ferroptosis susceptibility in cancer cells. Given prior reports suggesting that ZK53 targets ClpP (40), we sought to explore whether ClpP plays a functional role in the regulation of ferroptosis. Our findings confirm that ClpP is a crucial mediator of ferroptosis, and functional assays demonstrated that ZK53 promotes ferroptosis in a ClpP-dependent manner by disrupting mitochondrial function. Furthermore, *in vivo* studies revealed that ZK53 synergizes with the ferroptosis inducer IKE to trigger ferroptotic cell death in a mouse model without inducing measurable toxicity. These results suggest that targeting mitochondrial ClpP with ZK53 represents a promising strategy to enhance ferroptosis sensitivity in cancer cells, providing a novel therapeutic avenue for ferroptosis-based cancer treatment.

## Materials and methods

### Reagents

(1S,3R)-RSL3 (cat. HY-100218A), erastin (cat. HY-15763), and Mito-TEMPO (cat. HY-112879) were purchased from MedChemExpress (MCE). Liposomal 2000 Transfection Reagent (cat. 40802ES03), propidium iodide (cat. 40710ES03), and polybrene (cat. 40804ES76) were purchased from Yeasen. Puromycin (cat. ant-pr-1) was purchased from InvivoGen. SYTOX™ Green nucleic acid stain (cat. C1181S), GSH/GSSG Assay Kit (cat. S0053), JC-1 Mitochondrial Membrane Potential Assay Kit (cat. C2005), Blasticidin S HCl (cat. ST018), MPTP Assay Kit (cat. C2009S), and Lipid Peroxidation (MDA) Assay Kit (cat. S0131S) were purchased from Beyotime Biotechnology. RIPA Lysis Buffer (cat. AR0101 and AR0103) were purchased from Boster Biological Technology. Protease and Phosphatase Inhibitor Cocktail (cat. P002) was purchased from NCM Biotech. Bradford 1× Dye Reagent (cat. 5000205) was purchased from Bio-Rad. Cell Counting Kit-8 (cat. K1018) was purchased from APExBIO. BODIPY™ 581/591 C11 (cat. D3861) and MitoSOX™ Red mitochondrial superoxide indicator (cat. M36007) were purchased from Thermo Fisher Scientific. IKE (cat. HF3347) was purchased from Hi-Future, while ZK53 (cat. T80725) and Liproxstatin-1 (Lip-1; cat. T2376) were purchased from TargetMol.

### Cell culture

HT-1080, HeLa, HEK-293T, and HCT-116 cell lines were obtained from the Cell Bank of the Chinese Academy of Sciences. HT-1080, HeLa, and HEK-293T cells were cultured in DMEM Medium, while HCT-116 cells were cultured in McCoy’s 5A Medium (Meilunbio). All culture media were supplemented with 10% fetal bovine serum (ExCell Bio) and 1% penicillin-streptomycin (Gibco), and the cells were maintained at 37°C with 5% CO_2_ in a humidified incubator. All cell lines were regularly tested and confirmed to be free from mycoplasma contamination.

### Mitochondrial-targeted library screen

A mitochondrial-targeted library containing ~900 compounds was purchased from MedChemExpress (MCE) (Catalog number: HY-L089). HT-1080 cells were seeded into 96-well plates at a density of 6 × 10³ cells per well. On the second day, the cells were treated with 5 µM of compounds, which were co-incubated with either DMSO or RSL3 (100 nM). After 12 hours of incubation, cell viability was assessed using the CCK8 assay. Cell viability (%) was calculated using the following formula:


$$\text{Cell Viability}(\%)=\frac{\left({\text{OD}}_{\text{treated}}-{\text{OD}}_{\text{blank}}\right)}{\left({\text{OD}}_{\text{control}}-{\text{OD}}_{\text{blank}}\right)}\times 100$$

where OD represents the optical density at 450 nm for the treated group (OD_treated_), the control group (OD_control_), and the blank group (OD_blank_).

### Plasmid constructs and small interfering RNAs

The HA-tagged wild-type ClpP construct was generated by cloning the full-length ClpP cDNA into the pCDH vector. Short hairpin RNAs (shRNAs) targeting ClpP were cloned into the pLKO lentiviral vector. The following shRNA sequences were used: sh-ClpP:5’-GCCCATCCACATGTACATCAA-3’ and 5’-GCTCAAGAAGCAGCTCTATAA-3’.

All primers and shRNA sequences used in the experiments were synthesized by Tsingke Biotechnology. All plasmid constructs were verified by sequencing. Stable cell lines were selected and analyzed by Western blotting to confirm knockdown or overexpression efficiency.

### Lentivirus production and stable cell line construction

HEK-293T cells were seeded in 60 mm dishes and transfected with the plasmid of interest, psPAX2 packaging plasmid, and pMD2.G-VSV-G envelope plasmid at a 4:3:1 ratio using Liposomal Transfection Reagent (Yeasen) when cell confluence reached 90%. Fresh medium was replaced 6 hours post-transfection. The culture medium was collected 48 hours later, filtered through a 0.22 µm sterile filter, and supplemented with 10 μg/mL polybrene (Yeasen) for infection of HT-1080 cells. Lentivirus-transduced cells were selected for 48 hours with 2 μg/mL puromycin (Invitrogen) or 10 μg/mL blasticidin S HCl (BSD, Beyotime). Stable cell lines were confirmed by Western blotting.

### Western blot analysis

Cells were trypsinized, washed twice with ice-cold PBS, and lysed in lysis buffer (BOSTER) with a protease inhibitor cocktail (NCM Biotech). The supernatant was collected after centrifugation at 13,000 × g for 20 minutes. Protein concentration was measured using Bradford reagent (Bio-Rad). Protein were resolved by SDS-PAGE and transferred to PVDF membranes (Millipore). Membranes were blocked with 5% nonfat milk for 1 hour, then incubated with primary antibodies followed by HRP-conjugated goat secondary antibodies (Abclonal). Immunodetection was performed with an ECL kit (epizyme) and captured using a chemiluminescence imager (Bio-Rad).

The following blotting reagents and antibodies were used: Rabbit anti-β-Actin (cat. AC038, 1:20,000 for IB), anti-GAPDH (cat. A19056, 1:50,000 for IB), mouse anti-β-Tubulin (cat. AC021, 1:5,000 for IB), HRP-conjugated Goat anti-Mouse IgG (H+L) (cat. AS003, 1:5,000 for IB), HRP-conjugated Goat anti-Rabbit IgG (H+L) (cat. AS014, 1:5,000 for IB) antibodies were purchased from ABclonal. Rabbit anti-CLPP (cat. 15698-1-AP, 1:1,000 for IB), anti-HA tag (cat. 51064-2-AP, 1:5,000 for IB) antibodies were purchased from Proteintech. Mouse anti-GPX4 (cat.sc-166570, 1:500 for IB) antibodies were purchased from Santa Cruz.

### Cell viability assay

Cells were seeded in 96-well plates and cultured overnight at 37°C in a humidified atmosphere containing 5% CO_2_. The next day, cells were treated with the indicated drugs for the specified duration. The medium was replaced with PBS containing 10% CCK8 reagent (APEx BIO). After 1 hour of incubation at 37°C, absorbance at 450 nm was measured using a microplate reader (Thermo Scientific). The combination index (CI) for drug interactions was calculated according to the Chou-Talalay method (41). Specifically, a CI value of 1 indicates an additive interaction, CI values greater than 1 indicate antagonism, and CI values less than 1 reflect a synergistic interaction between the drugs.

### SYTOX fluorescence imaging

Cells were seeded in 96-well plates and cultured overnight at 37°C in a humidified atmosphere containing 5% CO_2_. The next day, cells were treated with the indicated drugs for the specified duration. Subsequently, cells were incubated with HBSS containing SYTOX™ Green nucleic acid stain (Beyotime) at 37°C for 30 min in the dark. Following incubation, cells were washed once with PBS and then replaced with fresh HBSS. Fluorescence images were acquired directly using an inverted fluorescence microscope equipped with a GFP channel (Ex/Em ≈ 488/500–550 nm).

### Cell morphology images

Cells were seeded in 96-well plates and cultured overnight at 37°C in a humidified incubator with 5% CO_2_. The following day, cells were treated with the indicated compounds for the specified durations. After treatment, cells were directly observed without washing to preserve their native morphology and treatment-associated features.

Cell morphology images were acquired using an inverted microscope equipped with a phase-contrast objective lens. Representative fields were selected and captured under identical imaging settings across all conditions to ensure consistency. The acquired images were used to visualize and document morphological changes induced by drug treatment.

### Cell death measurement

Cells were seeded in 6-well plates and cultured overnight at 37°C in a humidified atmosphere containing 5% CO_2_. The next day, cells were treated with the indicated drugs for the specified duration. After treatment, cells were stained with propidium iodide (PI) (Yeasen) at 37°C for 30 minutes. Cells were then trypsinized, washed, and resuspended in PBS. The fraction of dead cells was analyzed using a flow cytometer (Advanteon V6B5R3, Agilent), and data were processed with FlowJo software.

### Lipid peroxidation measurement

Cells were seeded in 6-well plates and cultured overnight at 37°C in a humidified atmosphere containing 5% CO_2_. The next day, cells were treated with the indicated drugs for the specified duration. The cells were stained with 5 µM BODIPY™ 581/591 C11 (Thermo Scientific) at 37°C for 30 minutes. After trypsinization, the cells were washed and resuspended in PBS. Lipid peroxidation levels were analyzed using a flow cytometer (Advanteon V6B5R3, Agilent) and data were analyzed using FlowJo software.

### Determination of GSH levels

Cells were seeded onto 100 mm dishes and cultured overnight at 37°C in a humidified atmosphere containing 5% CO_2_. The next day, cells were treated with the indicated drugs for the specified duration. Cells were collected for GSH and GSSG measurement using GSH and GSSG Assay Kit (Beyotime) according to the manufacturer’s protocol. The GSH and GSSG concentrations were calculated using a standard curve and normalized to the total protein level.

### Mitochondrial membrane potential measurement

Cells were seeded in 6-well plates and cultured overnight at 37°C in a humidified atmosphere containing 5% CO_2_. The next day, cells were treated with the indicated drugs for the specified duration. The cells were stained with JC-1 (Beyotime) at 37°C for 30 minutes. After trypsinization, the cells were washed and resuspended in PBS. Mitochondrial membrane potential was analyzed using a flow cytometer (Advanteon V6B5R3, Agilent) and data were processed with FlowJo software.

### Mitochondrial ROS measurement

Cells were seeded in 6-well plates and cultured overnight at 37°C in a humidified atmosphere containing 5% CO_2_. The next day, cells were treated with the indicated drugs for the specified duration. The cells were stained with 5 µM MitoSOX (Invitrogen) at 37°C for 30 minutes. After trypsinization, the cells were washed and resuspended in PBS. Mitochondrial ROS levels were analyzed using a flow cytometer (Advanteon V6B5R3, Agilent) and data were processed with FlowJo software.

### Mitochondrial permeability transition pore measurement

Cells were seeded in 6-well plates and cultured overnight at 37°C in a humidified atmosphere containing 5% CO_2_. The next day, cells were treated with the indicated drugs for the specified duration. The cells were stained using the MPTP Assay Kit (Beyotime) according to the manufacturer’s instructions. After trypsinization, the cells were washed and resuspended in PBS. Mitochondrial permeability transition pore levels were analyzed using a flow cytometer (Advanteon V6B5R3, Agilent) and data were processed with FlowJo software.

### Animal treatment

This study was conducted in compliance with all relevant ethical guidelines. All animal procedures were approved by the Institutional Animal Care and Use Committee of Zhejiang University and were carried out in accordance with its established protocols, under the ethical approval number ZJU20250193. Four-week-old female, specific pathogen-free (SPF) BALB/c nude mice were obtained from Shanghai SLAC Laboratory Animal Co., Ltd. (Shanghai, China). The mice were provided with standard rodent chow and water ad libitum and were housed in a specific pathogen-free environment with a 12-hour light/12-hour dark cycle.

For the xenograft tumor assay, 6 × 10^6^ HCT-116 cells were subcutaneously injected into the flanks of six-week-old female BALB/c nude mice. When tumors reached a size of 50–100 mm³, the mice were randomly assigned to different treatment groups and administered either vehicle, IKE (25 mg/kg; HI-FUTURE), ZK53 (20 mg/kg; TargetMol), or Lip-1 (10 mg/kg; TargetMol) via intraperitoneal injection every other day. The vehicle consisted of 10% dimethyl sulfoxide (DMSO), 40% polyethylene glycol 300 (PEG300), 5% Tween-80, and 45% saline (v/v/v/v), which served as a common solvent system for all compounds. The maximum allowable tumor burden, as per the ethics committee, was 1,500 mm³, and this threshold was not surpassed during the study.

### MDA levels measurement

MDA levels were measured using a lipid peroxidation MDA assay kit (Beyotime). Briefly, fresh tumor tissues were lysed with RIPA buffer (BOSTER). The supernatant was incubated with thiobarbituric acid (TBA) at 100°C for 15 minutes to form MDA-TBA adducts. After centrifugation, the absorbance of the supernatant at 532 nm was measured using a microplate reader (Thermo Scientific). MDA levels were normalized to protein concentration.

### Histological analysis

Fresh tissues were fixed in 4% paraformaldehyde for 48 hours, dehydrated, and embedded in paraffin. Five-micrometer sections were cut for immunohistochemistry (IHC). Sections were incubated overnight at 4°C with primary antibodies against Ki-67 (1: 300, ab16667, Abcam) and 4-HNE (1:400, ab46545, Abcam). Images were captured from five random regions per tumor at 200× magnification using an EVOS M7000 microscope (Invitrogen). The expression of Ki-67 and 4-HNE was quantified using ImageJ software by measuring the integrated optical density (IOD) of positive staining in each section.

### Quantifications and statistical analysis

All experiments were performed with at least three independent replicates. Statistical analyses were conducted using GraphPad Prism V8. Data are presented as mean ± SD. Statistical significance was assessed using an unpaired Student’s t-test, one-way ANOVA, or two-way ANOVA, as appropriate. A p-value of < 0.05 was considered statistically significant.

## Results

### High-throughput screening identifies that ZK53 enhances cellular vulnerability to RSL3 treatment

**Figure 1:**
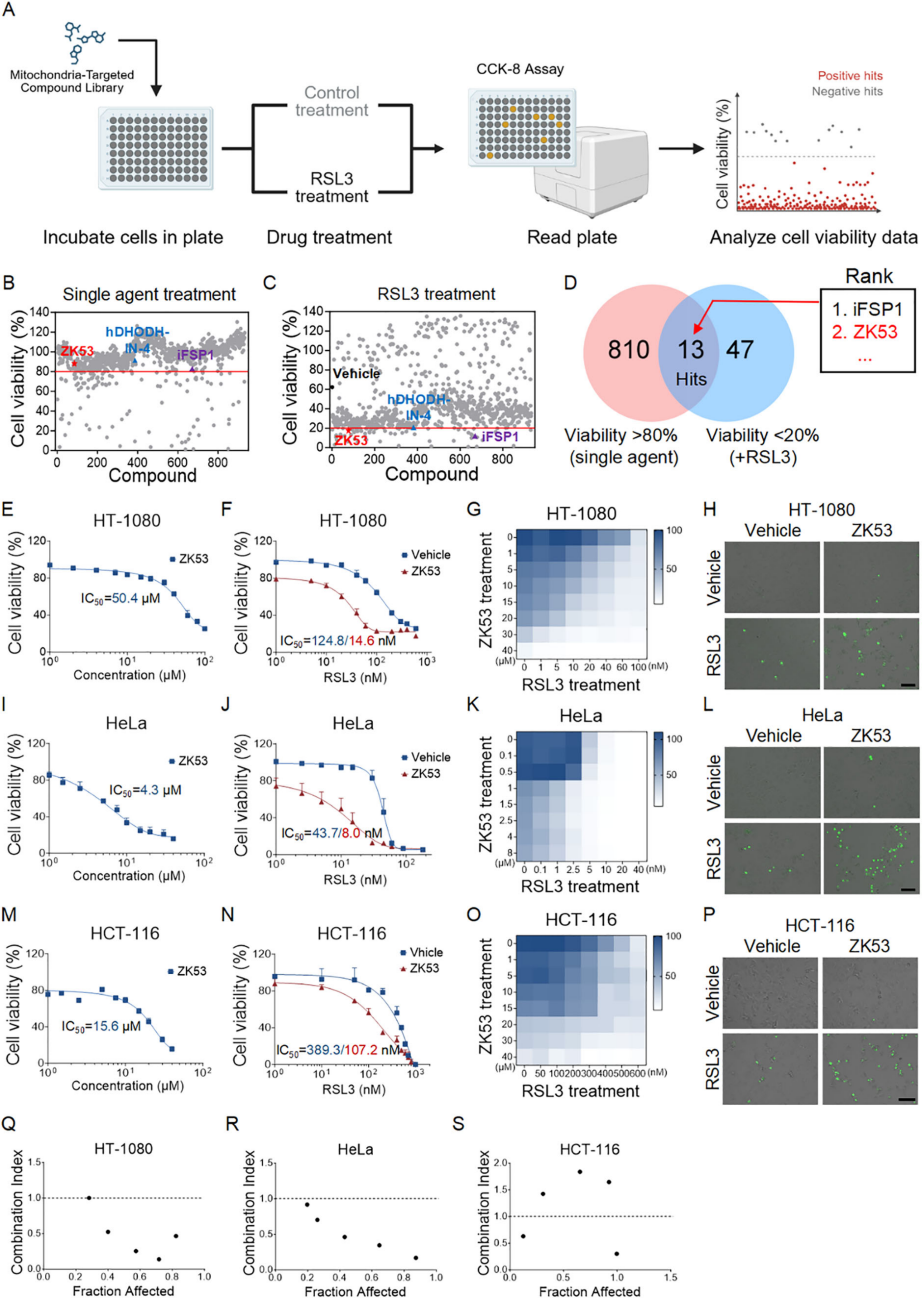
High-throughput screening identifies that ZK53 enhances cellular vulnerability to RSL3 treatment. **(A)** Schematic representation of high-throughput screening for ferroptosis-sensitizing compounds from a mitochondrial-targeted drug library. **(B, C)** Effects of individual treatment **(B)** or co-treatment with 100 nM RSL3 **(C)** on the viability of HT-1080 cells after 12 h. Compounds showing cell viability greater than 80% in **(B)** and less than 20% in **(C)** were considered candidate ferroptosis-sensitizing compounds. Previously reported positive control compounds are marked by blue and purple triangles, while the positive compound identified in this study is indicated by red pentagrams. Vehicle-treated controls are shown as black circles. **(D)** Venn diagram showing 13 positive compounds identified in the screening, among which ZK53 ranked within the top two hits. **(E, I, M)** Cell viability of HT-1080 **(E)**, HeLa **(I)**, and HCT-116 **(M)** cells after 12 h of treatment with different concentrations of ZK53. **(F)** Cell viability of HT-1080 cells after 12-h treatment with different concentrations of RSL3 in the presence of 10 µM ZK53. **(J)** Cell viability of HeLa cells after 12-h treatment with different concentrations of RSL3 in the presence of 1 µM ZK53. **(N)** Cell viability of HCT-116 cells after 12-h treatment with different concentrations of RSL3 in the presence of 5 µM ZK53. **(G, K, O)** Heatmaps of cell viability for HT-1080 **(G)**, HeLa **(K)**, and HCT-116 **(O)** cells following 12 h of treatment with different concentrations of ZK53 and RSL3. **(H)** Sytox staining of HT-1080 cells after a 12-h treatment with 100 nM RSL3 and 10 µM ZK53, scale bar: 100 µm. **(L)** Sytox staining of HeLa cells after a 12-h treatment with 50 nM RSL3 and 1 µM ZK53, scale bar:100 µm. **(P)** Sytox staining of HCT-116 cells after a 12-h treatment with 200 nM RSL3 and 5 µM ZK53, scale bar:100 µm. **(Q-S)** The combination index (CI) values for the combination of ZK53 and RSL3 in HT-1080 **(Q)**, HeLa **(R)**, and HCT-116 **(S)** cells were determined using Calcusyn software. Data were presented as the mean ± SD.; n = 3 biologically independent experiments.

To identify potential ferroptosis sensitizers, we conducted a high-throughput screening of a mitochondrial-targeted drug library using the HT-1080 cell line, a well-established model for ferroptosis research. Our goal was to discover compounds that could enhance ferroptosis induced by RSL3, while minimizing general cytotoxicity (**Figure 1A**). This was accomplished by comparing the effects of individual compounds (with cell viability >80%) to the combined treatments with RSL3, where the single RSL3 treatment yielded ~60% cell viability, resulting in cell viability <20% when combined.

Among the identified compounds, we confirmed several inhibitors targeting known ferroptosis-related regulators, including the DHODH-targeting inhibitor hDHODH-IN-4 and the FSP1-targeting inhibitor iFSP1, the targets of which, DHODH (17) and FSP1 (42, 43), have been previously implicated in ferroptosis regulation (**Figures 1B-D**). These findings validate the robustness and reliability of our screening strategy for identifying ferroptosis sensitizers. Notably, in addition to these compounds targeting previously characterized modulators, we identified ZK53 as a novel hit that significantly increased cellular susceptibility to RSL3 (**Figures 1B-D**).

To further validate our screening results, we recharacterized the identified compound ZK53 in different cell lines to ensure that its ferroptosis-sensitizing effect was reproducible and not cell line–specific. First, we assessed its cytotoxicity across a range of concentrations. Cell viability assays indicated that low concentrations of ZK53 (below 15 µM for HT-1080 cells, below 1 µM for HeLa cells, and below 10 µM for HCT-116 cells) did not significantly affect cell viability (**Figures 1E, I, M**). However, at higher concentrations, ZK53 partially inhibited cell viability, which is consistent with previous reports suggesting its potential impact on cell proliferation (40). To minimize cytotoxic effects and ensure the consistency of subsequent experiments, we selected non-toxic concentration of 10 µM for HT-1080 cells, 1 µM for HeLa cells, and 5 µM for HCT-116 cells for all subsequent studies.

Next, we validated the sensitizing effect of ZK53 on RSL3. Cell viability assays, using both concentration gradients of the drug combination and individual agents, demonstrated that ZK53 significantly enhanced RSL3-induced cell death across different cell lines (**Figures 1F, G, J, K, N, O**). The drug interaction between ZK53 and RSL3 was assessed by calculating the combination index values (**Figures 1Q-S**), which revealed a significant synergistic effect of ZK53 when combined with RSL3. Similar results were obtained through Sytox staining, further confirming the enhanced cell death induced by the combination treatment (**Figures 1H, L, P**).

In sum, these results suggest that ZK53 may serve as a promising candidate for therapeutic strategies targeting ferroptosis.

### ZK53 increases cellular vulnerability to ferroptosis

Given that RSL3 targets not only GPX4 but also other potential targets (44), we conducted further investigations to confirm that the sensitizing effect of ZK53 on RSL3-induced cell death is mediated via ferroptosis.

**Figure 2:**
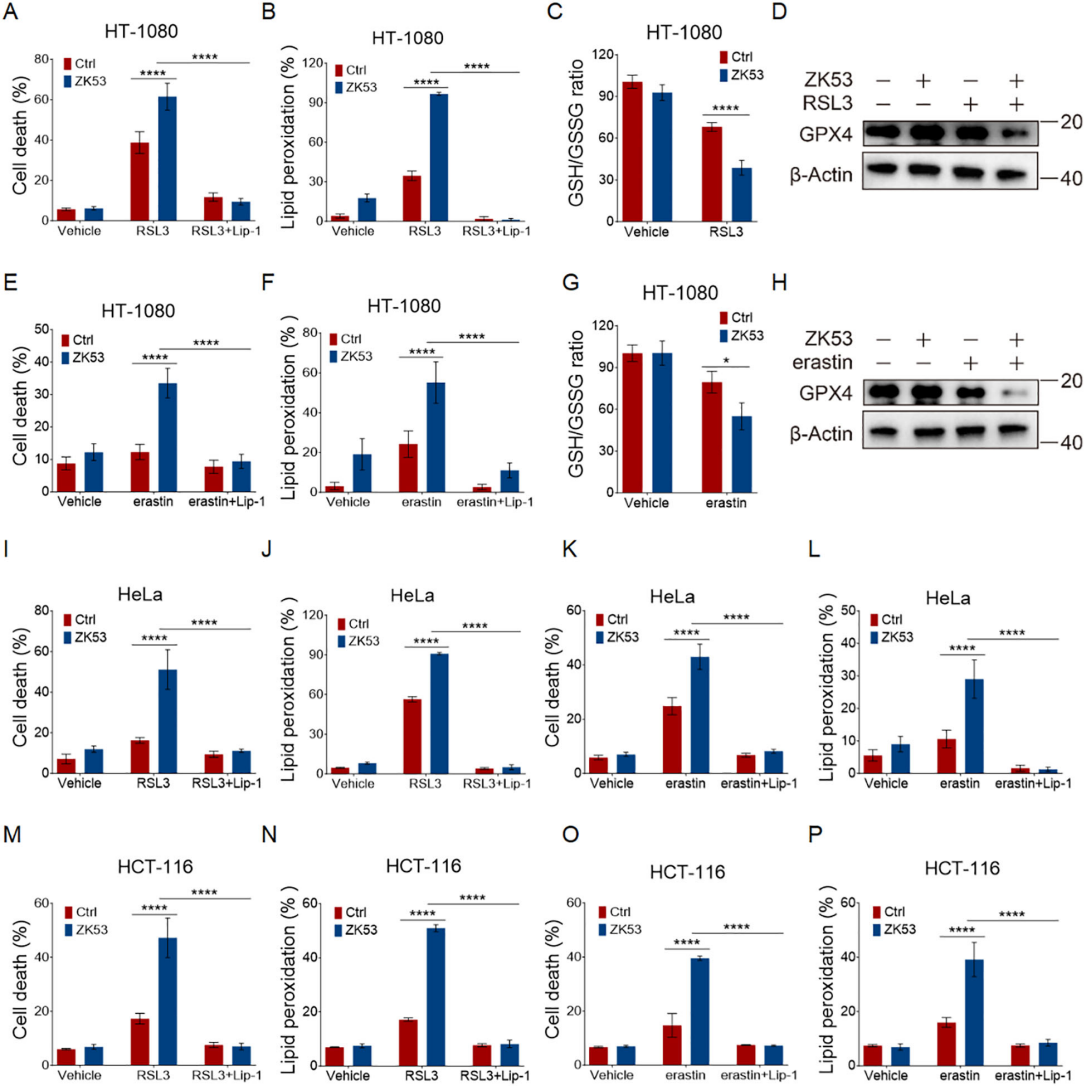
ZK53 increases cellular vulnerability to ferroptosis. **(A, E)** Cell death measurements of HT-1080 cells treated with 100 nM RSL3 **(A)** for 12 h and 2.5 µM erastin **(E)** for 24 h following a 0.5-h pretreatment with 2 µM Lip-1, with or without 10 µM ZK53 treatment as indicated. **(B, F)** Lipid peroxidation measurements of HT-1080 cells treated with 100 nM RSL3 **(B)** for 4 h and 2.5 µM erastin **(F)** for 12 h following a 0.5-h pretreatment with 2 µM Lip-1, with or without 10 µM ZK53 treatment as indicated. **(C, G)** GSH measurements of HT-1080 cells treated with 100 nM RSL3 **(C)** for 4 h and 2.5 µM erastin **(G)** for 12 h, with or without 10 µM ZK53 treatment as indicated. **(D, H)** Western blot analysis of GPX4 protein levels in HT-1080 cells treated with 100 nM RSL3 for 4 h **(D)** or 2.5 µM erastin for 12 h **(H)**, with or without 10 µM ZK53 treatment as indicated. **(I, K)** Cell death measurements of HeLa cells treated with 50 nM RSL3 **(I)** for 12 h and 10 µM erastin **(K)** for 24 h following a 0.5-h pretreatment with 2 µM Lip-1, with or without 1 µM ZK53 treatment as indicated. **(J, L)** Lipid peroxidation measurements of HeLa cells treated with 50 nM RSL3 **(J)** for 6 h and 10 µM erastin **(L)** for 12 h following a 0.5-h pretreatment with 2 µM Lip-1, with or without 1 µM ZK53 treatment as indicated. **(M, O)** Cell death measurements of HCT-116 cells treated with 200 nM RSL3 **(M)** for 12 h and 20 µM erastin **(O)** for 24 h following a 0.5-h pretreatment with 2 µM Lip-1, with or without 5 µM ZK53 treatment as indicated. **(N, P)** Lipid peroxidation measurements of HCT-116 cells treated with 200 nM RSL3 **(N)** for 6 h and 20 µM erastin **(P)** for 12 h following a 0.5-h pretreatment with 2 µM Lip-1, with or without 5 µM ZK53 treatment as indicated. Data were presented as the mean ± SD; n = 3 biologically independent experiments. Statistical analysis was performed using two-way ANOVA. *p < 0.05, **p < 0.01, ***p < 0.001, ****p < 0.0001, NS, not significant.

In HT-1080 cells, the classical ferroptosis inhibitor Lip-1 completely abolished the sensitizing effect of ZK53 on RSL3-induced cell death (**Figure 2A**). Lipid peroxidation, a key hallmark of ferroptosis, was assessed using the lipid peroxidation sensor BODIPY staining. The results showed that ZK53 significantly enhanced RSL3-induced lipid peroxidation, an effect that was fully reversed by Lip-1 (**Figure 2B**). Consistently, the intracellular GSH/GSSG ratio, serving as a quantitative indicator of the redox equilibrium (45, 46), and GPX4 protein expression, representing the cellular antioxidant defense against lipid peroxide accumulation (2, 47), were both significantly reduced following combined treatment with ZK53 and RSL3 (**Figures 2C, D**). In parallel experiments, we employed another ferroptosis inducer, erastin, and obtained similar results (**Figures 2E–H**). These findings were further validated in HeLa (**Figures 2I–L**) and HCT-116 cells (**Figures 2M–P**).

Together, our data confirm that ZK53 synergizes with ferroptosis inducers such as RSL3 and erastin to promote ferroptosis, supporting its potential as a therapeutic sensitizer for ferroptosis-based treatments.

### ClpP deficiency inhibits ferroptosis

Having established that ZK53 sensitizes cells to ferroptosis, and given that ZK53 is a selective inhibitor of the human Clp protease subunit (HsClpP) (40), we next explored the potential involvement of ClpP in the regulation of ferroptosis. ClpP plays a critical role in mitochondrial homeostasis and stress responses by degrading misfolded or damaged proteins, especially under oxidative stress conditions (36, 48–50).

**Figure 3:**
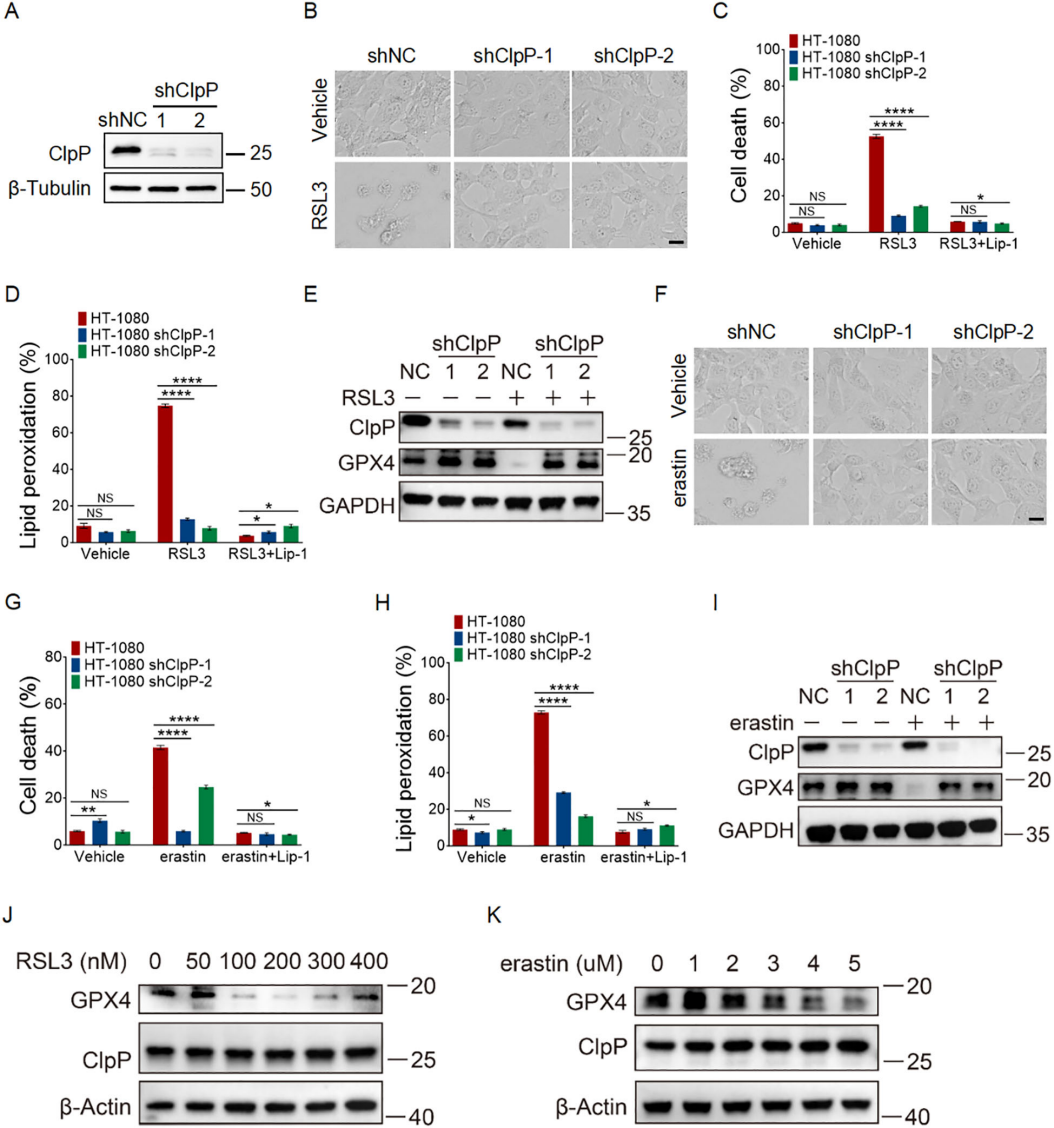
ClpP deficiency inhibits ferroptosis. **(A)** ClpP-knockdown cell lines were generated using the RNAi system and verified by western blotting. **(B, F)** Cell morphology images of control and ClpP-knockdown HT-1080 cells treated with 200 nM RSL3 for 12 h **(B)** or 5 µM erastin for 24 h **(F)**, following pretreatment with 2 µM Lip-1 for 0.5 h, scale bar: 20 µm. **(C, G)** Cell death measurements of control and ClpP knockdown HT-1080 cells treated with 200 nM RSL3 **(C)** for 12 h and 5 µM erastin **(G)** for 24 h, following pretreatment with 2 µM Lip-1 for 0.5 h. **(D, H)** Lipid peroxidation measurements of control and ClpP knockdown HT-1080 cells treated with 200 nM RSL3 **(D)** for 4 h and 5 µM erastin **(H)** for 12 h, following pretreatment with 2 µM Lip-1 for 0.5 h. **(E, I)** Western blot analysis of GPX4 protein levels in control and ClpP knockdown HT-1080 cells treated with 200 nM RSL3 **(E)** for 4 h and 5 µM erastin **(I)** for 12 h, following pretreatment with 2 µM Lip-1 for 0.5 h. **(J, K)** Western blot analysis of ClpP and GPX4 protein levels in HT-1080 cells treated with RSL3 for 4 h **(J)** or erastin for 12 h **(K)**. Data were presented as the mean ± SD.; n = 3 biologically independent experiments. Statistical analysis was performed using two-way ANOVA. *p < 0.05, **p < 0.01, ***p < 0.001, ****p < 0.0001, NS, not significant.

We hypothesized that ClpP might contribute to the cellular mechanisms underlying ferroptosis. To investigate this, we performed functional studies in HT-1080 cells with ClpP knockdown using specific shRNA (**Figure 3A**). Cell morphology, cell death, lipid ROS levels, and GPX4 protein expression were examined in both control and ClpP-knockdown HT-1080 cells.

ClpP depletion significantly attenuated cell death induced by both RSL3 and erastin (**Figures 3B, C, F, G**). Consistently, knockdown of ClpP markedly inhibited lipid peroxidation and preserved GPX4 protein levels in response to RSL3 and erastin treatment (**Figures 3D, E, H, I**).

To determine whether ferroptosis inducers affect ClpP expression itself, we assessed ClpP protein levels in cells treated with RSL3 or erastin. As shown in **Figures 3J, K**, ClpP expression remained largely unchanged after treatment with either compound. These findings indicate that RSL3 and erastin do not regulate ClpP expression directly. Instead, the observed synergistic effect of ZK53 with RSL3 is likely mediated through ClpP’s functional involvement in ferroptosis rather than changes in its abundance.

### Overexpression of wild-type and hyperactivated ClpP promotes ferroptosis

**Figure 4:**
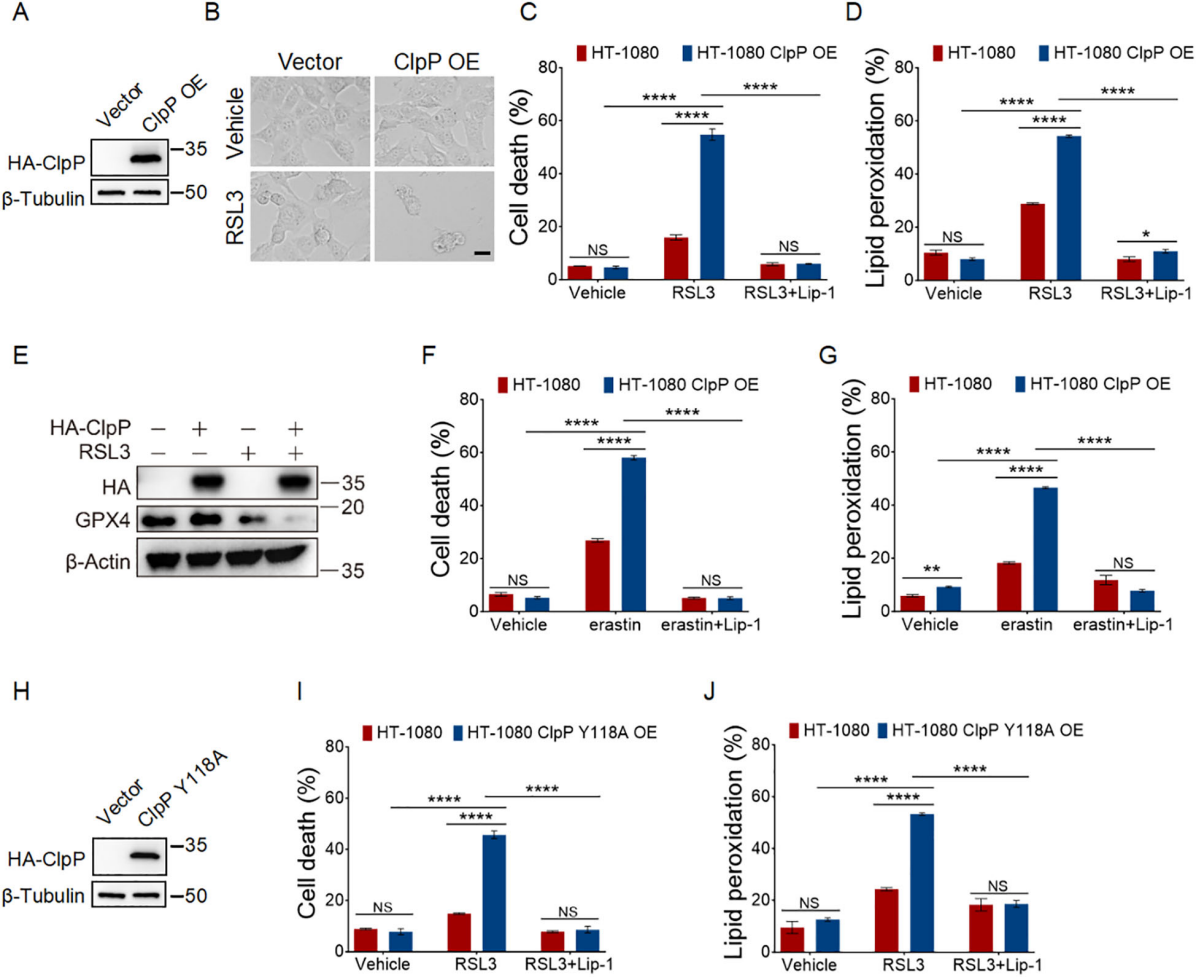
Overexpression of wild-type and hyperactivated ClpP promotes ferroptosis. **(A)** ClpP overexpressed HT-1080 cells were generated and verified by western blotting. **(B)** Cell morphology images of control and ClpP wild-type overexpressed HT-1080 cells treated with 100 nM RSL3 for 12 h, scale bar: 20 µm. **(C, F)** Cell death measurements of control and ClpP wild-type overexpressed HT-1080 cells treated with 100 nM RSL3 **(C)** for 12 h and 2.5 µM erastin **(F)** for 24 h, following pretreatment with 2 µM Lip-1 for 0.5 h. **(D, G)** Lipid peroxidation measurements of control and ClpP wild-type overexpressed HT-1080 cells treated with 100 nM RSL3 **(D)** for 4 h and 2.5 µM erastin **(G)** for 12 h, following pretreatment with 2 µM Lip-1 for 0.5 h. **(E)** Western blot analysis of GPX4 protein levels in control and ClpP wild-type overexpressed HT-1080 cells treated with 100 nM RSL3 for 4 h. **(H)** ClpP mutant (Y118A) overexpressed HT-1080 cells were generated and verified by western blotting. **(I)** Cell death measurements of control and ClpP Y118A mutant overexpressed HT-1080 cells treated with 100 nM RSL3 for 12 h, following pretreatment with 2 µM Lip-1 for 0.5 h. **(J)** Lipid peroxidation measurements of control and ClpP Y118A mutant overexpressed HT-1080 cells treated with 100 nM RSL3 for 4 h, following pretreatment with 2 µM Lip-1 for 0.5 h. Data were presented as the mean ± SD; n = 3 biologically independent experiments. Statistical analysis was performed using two-way ANOVA. *p < 0.05, **p < 0.01, ***p < 0.001, ****p < 0.0001, NS, not significant.

To further confirm the role of ClpP in ferroptosis, we overexpressed ClpP in HT-1080 cells (**Figure 4A**). ClpP overexpression potentiated RSL3- and erastin-induced ferroptosis, as evidenced by increased cell death and lipid peroxidation, along with a concomitant decrease in GPX4 protein levels (**Figures 4B–G**). These effects were completely abrogated by the ferroptosis inhibitor Lip-1 (**Figures 4C, D, F, G**).

Building on these findings, we next examined whether hyperactivation of ClpP could also enhance ferroptosis. The Y118A mutation in ClpP, a gain-of-function mutation, increases its protease activity and leads to hyperactivation of ClpP’s proteolytic function (51, 52). Overexpressing ClpP Y118A in HT-1080 cells resulted in significantly increased sensitivity to RSL3-induced cell death and lipid peroxidation compared to control cells (**Figures 4H-J**).

Together, these results further confirm that ClpP and its proteolytic activity play an important role in promoting ferroptosis.

### ZK53 sensitizes cells to ferroptosis via ClpP hyperactivation

While previous studies have established that ZK53 acts as a ClpP activator—identified through screening and later optimized for its potency (40)—it remains possible that ZK53 could promote ferroptosis through pathways independent of ClpP.

**Figure 5:**
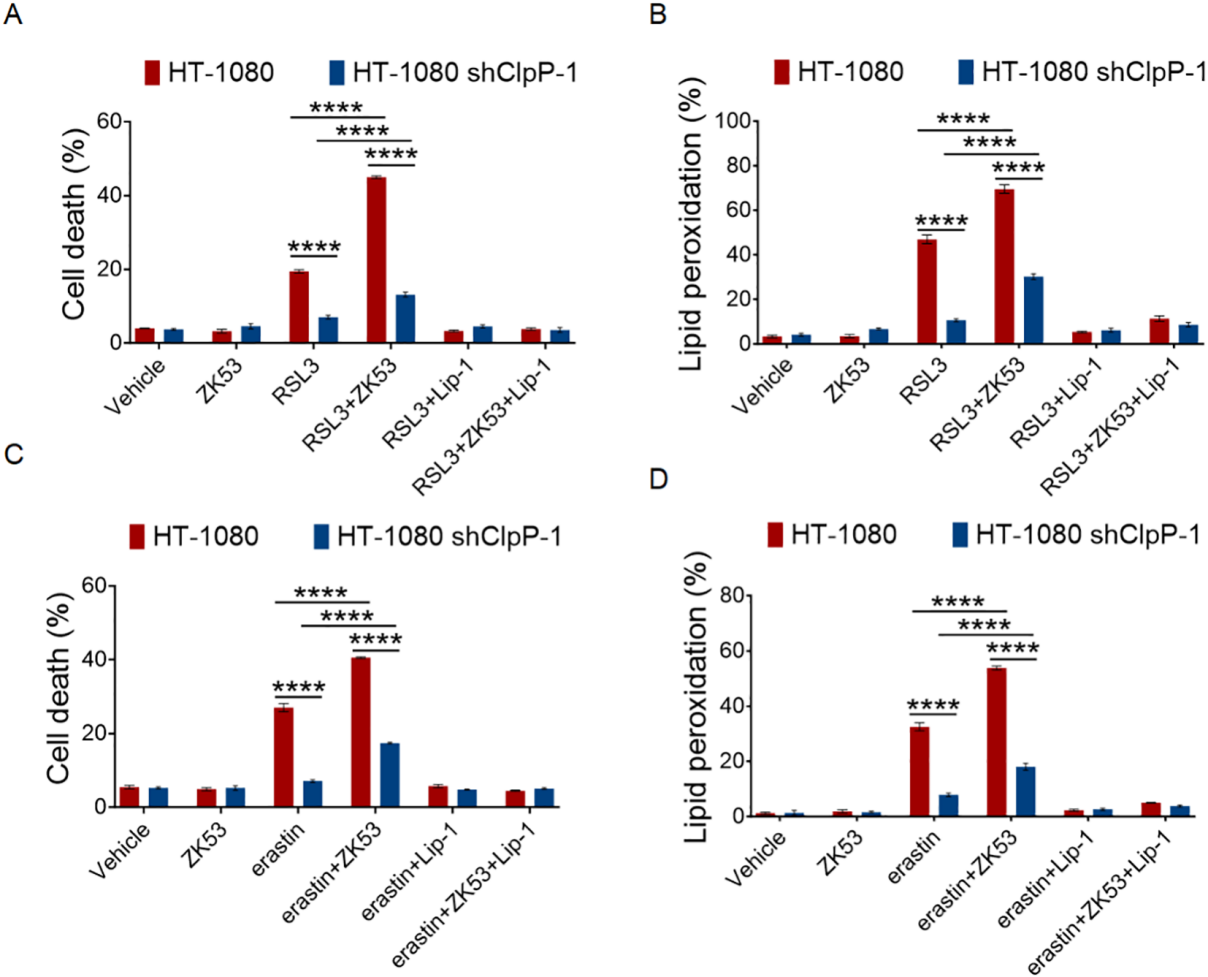
ZK53 sensitizes cells to ferroptosis via ClpP hyperactivation. **(A, C)** Cell death measurements of control and ClpP knockdown HT-1080 cells treated with 100 nM RSL3 **(A)** for 12 h and 2.5 µM erastin **(C)** for 24 h following a 0.5-h pretreatment with 2 µM Lip-1, with or without 10 µM ZK53 treatment as indicated. **(B, D)** Lipid peroxidation measurements of control and ClpP knockdown HT-1080 cells treated with 100 nM RSL3 **(B)** for 4 h and 2.5 µM erastin **(D)** for 12 h following a 0.5-h pretreatment with 2 µM Lip-1, with or without 10 µM ZK53 treatment as indicated. Data were presented as the mean ± SD; n = 3 biologically independent experiments. Statistical analysis was performed using two-way ANOVA. *p < 0.05, **p < 0.01, ***p < 0.001, ****p < 0.0001, NS, not significant.

To explore whether the ferroptosis-sensitizing effect of ZK53 is reliant on ClpP, we treated both wild-type and ClpP-deficient HT-1080 cells with ZK53, in combination with ferroptosis inducers. Our analysis of cell death and lipid peroxidation showed that ZK53’s ability to enhance RSL3-induced ferroptosis was significantly diminished in ClpP-deficient cells compared to wild-type controls (**Figures 5A, B**). Similarly, when we used erastin as an alternative ferroptosis inducer, the sensitizing effects of ZK53 on both cell death and lipid peroxidation were also notably reduced in the absence of ClpP (**Figures 5C, D**).

Although the ferroptosis-sensitizing effect of ZK53 was substantially attenuated by ClpP knockdown, a modest but significant residual enhancement persisted compared with treatment with RSL3 or erastin alone. This remaining activity may result from incomplete suppression of ClpP or potential ClpP-independent effects of ZK53.

Together, these findings indicate that the sensitizing effect of ZK53 on ferroptosis is primarily mediated through ClpP, and this mechanism is not specific to the choice of ferroptosis inducer.

### ZK53 amplify ferroptosis through mitochondrial dysfunction

Activation of ClpP has been shown to disrupt mitochondrial protein homeostasis, affecting critical mitochondrial components, including electron transport chain (ETC) subunits and TCA cycle enzymes like aconitase (ACO2) (51, 53). Given the pivotal role of mitochondrial dysfunction in ferroptosis, we hypothesized that ZK53 might amplify ferroptosis through its effects on mitochondrial integrity.

**Figure 6:**
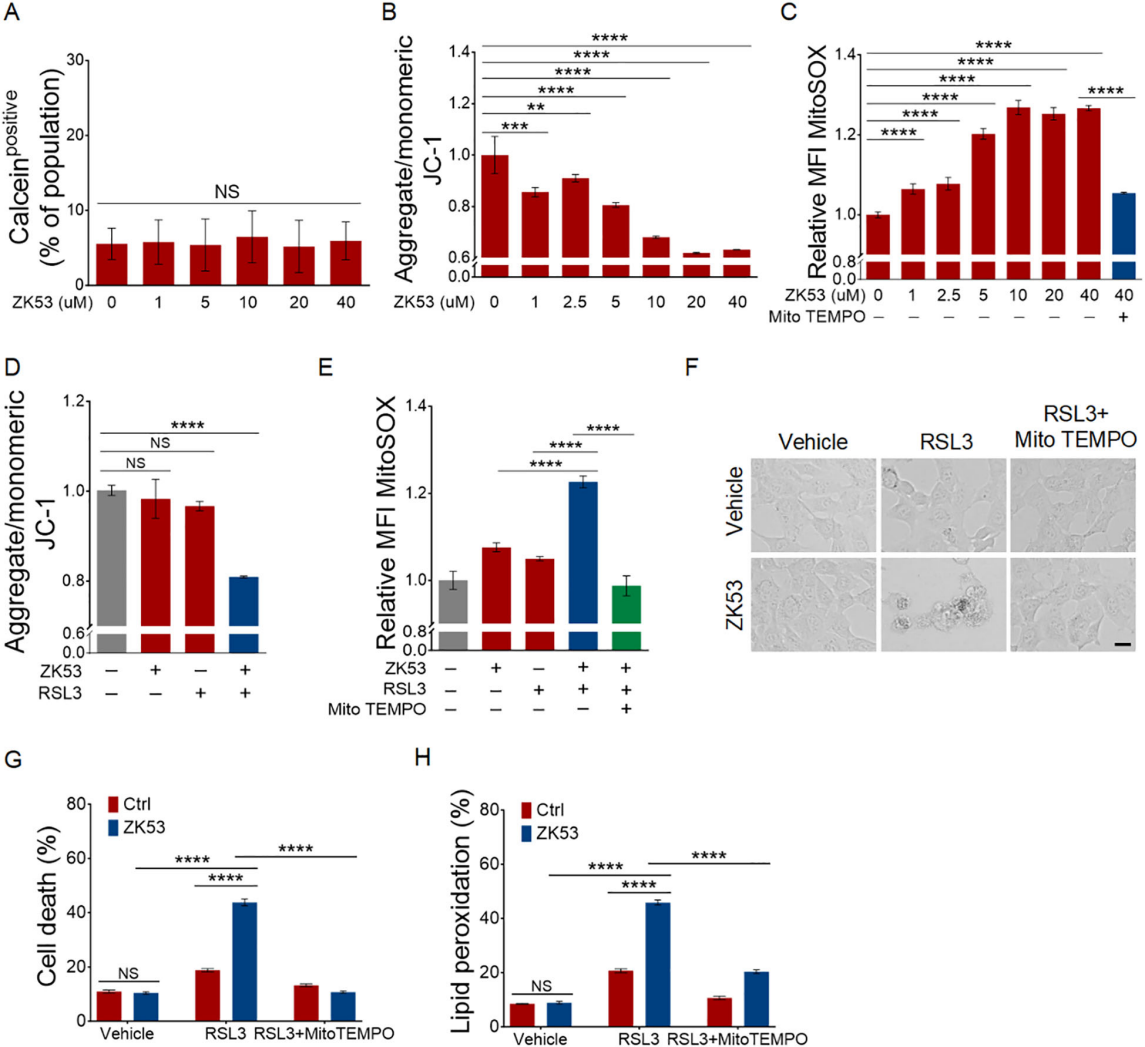
ZK53 induces ferroptosis through mitochondrial dysfunction. **(A)** Mitochondrial Permeability Transition Pore (mPTP) assay in HT-1080 cells after 12-h treatment with varying concentrations of ZK53, using calcein fluorescence as an indicator. **(B)** Mitochondrial membrane potential assay in HT-1080 cells after 12-h treatment with different concentrations of ZK53, using the JC-1 probe. **(C)** Mitochondrial ROS assay using MitoSOX in HT-1080 cells after 12-h treatment with varying concentrations of ZK53, with 10 µM mito-TEMPO as a mitochondrial ROS scavenger. **(D)** Mitochondrial membrane potential assay in HT-1080 cells after 12-h treatment with 1 µM ZK53 and/or 100 nM RSL3, using the JC-1 probe. **(E)** Mitochondrial ROS assay using MitoSOX in HT-1080 cells after 12-h treatment with 1 µM ZK53 and/or 100 nM RSL3, with 10 µM mito-TEMPO as a mitochondrial ROS scavenger. **(F)** Cell morphology images of HT-1080 cells after 12-h treatment with 10 µM ZK53 and/or 100 nM RSL3, using 10 µM mitoTEMPO as a mitochondrial ROS scavenger, scale bar: 20 µm. **(G)** Cell death measurements of HT-1080 cells after 12-h treatment with 10 µM ZK53 and/or 100 nM RSL3, using 10 µM mitoTEMPO as a mitochondrial ROS scavenger. **(H)** Lipid peroxidation measurements of HT-1080 cells after 4-h treatment with 10 µM ZK53 and/or 100 nM RSL3, using 10 µM mito-TEMPO as a mitochondrial ROS scavenger. Data were presented as the mean ± SD; n = 3 biologically independent experiments. Statistical analysis was performed using two-way ANOVA. *p < 0.05, **p < 0.01, ***p < 0.001, ****p < 0.0001, NS, not significant.

To test this hypothesis, we first assessed key mitochondrial function indicators in HT-1080 cells following ZK53 treatment. Although no significant changes were observed in mitochondrial permeability transition pore (MPTP) opening (**Figure 6A**), ZK53 treatment caused a concentration-dependent decrease in mitochondrial membrane potential (ΔΨm) (**Figure 6B**), as indicated by JC-1 staining. Additionally, MitoSOX staining showed a marked, concentration-dependent increase in mitochondrial ROS production, which was effectively mitigated by the mitochondrial ROS scavenger MitoTEMPO (**Figure 6C**). These findings suggest that ZK53 treatment disrupts mitochondrial normal function.

We then examined whether co-treatment with ZK53 and the ferroptosis inducer RSL3 would further exacerbate mitochondrial dysfunction. Indeed, combined treatment led to a more pronounced reduction in mitochondrial membrane potential, and a further increase in mitochondrial ROS levels compared to treatment with either agent alone (**Figures 6D, E**). This synergistic effect suggests that ZK53 and RSL3 collaborate to amplify mitochondrial damage.

To further validate the role of mitochondrial dysfunction in ZK53-induced ferroptosis, we examined whether mitochondrial ROS scavengers could attenuate the ferroptosis-sensitizing effect of ZK53. Treatment with MitoTEMPO markedly mitigated the enhancement of RSL3-induced ferroptotic cell death and lipid peroxidation caused by ZK53 (**Figures 6F–H**), as evidenced by cell morphology imaging, PI staining, and lipid peroxidation assays. These results demonstrate that mitochondrial ROS are essential mediators of the ZK53/ClpP-dependent ferroptosis amplification, supporting the critical role of mitochondrial dysfunction in ZK53-induced ferroptotic signaling.

In conclusion, our data demonstrate that ZK53 promotes ferroptosis through mitochondrial dysfunction, with mitochondrial ROS generation serving as a key driver of this process.

### ZK53 enhances tumor sensitivity to ferroptosis *in vivo*

Targeting ferroptosis has emerged as a promising strategy in cancer therapy, but the effectiveness of small-molecule inducers is often limited by resistance mechanisms. Consequently, combination therapies have become a key focus of research to enhance ferroptosis-based treatments (8).

**Figure 7:**
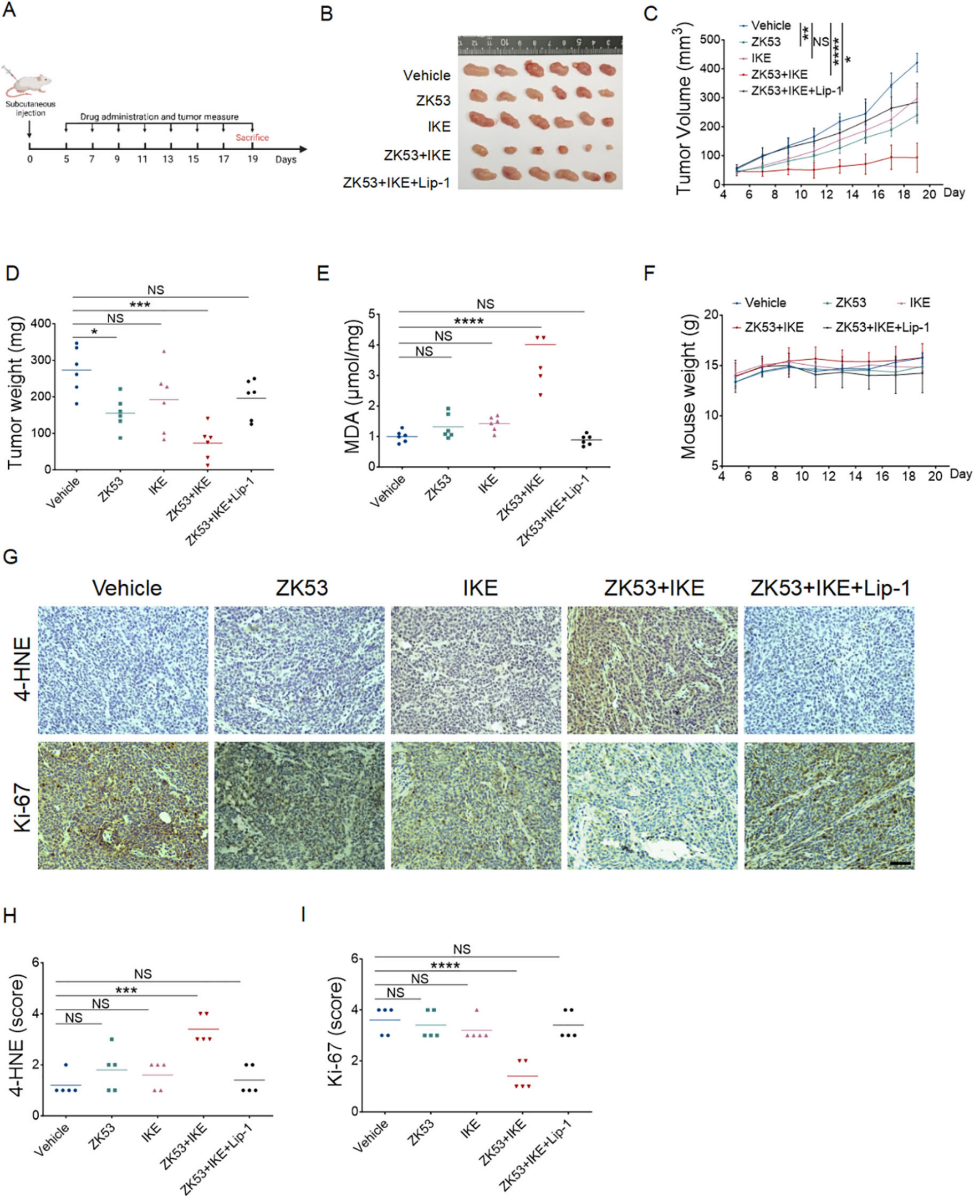
ZK53 enhances tumor sensitivity to ferroptosis *in vivo.***(A)** Schematic representation of the experimental design for the subcutaneous tumor model in nude mice. **(B)** Images of HCT-116 xenograft tumors excised from mice after the final assessment of tumor volumes. **(C)** Tumor volume was measured every two days, and statistical significance was determined based on the difference in tumor volume on the final day of measurement. **(D, E)** Average tumor weights **(D)** and relative MDA levels **(E)** of the excised HCT-116 tumors. **(F)** Body weight was measured every two days. **(G)** Representative images of 4-HNE and Ki-67 staining from tumors, scale bar: 100 µm. **(H, I)** 4-HNE **(H)** and Ki-67 **(I)** immunohistochemistry scoring of the excised HCT-116 tumors. Data were presented as the mean ± SEM.; n ≥ 5 biologically independent samples. Statistical analysis was performed using two-way ANOVA. *p < 0.05, **p < 0.01, ***p < 0.001, ****p < 0.0001, NS, not significant.

Given the significant ferroptosis-sensitizing effect of ZK53 observed *in vitro*, we further assessed its therapeutic potential *in vivo*. To this end, we established a xenograft tumor model by subcutaneously implanting HCT-116 cells (a ferroptosis-resistant cell line) into BALB/c nude mice, followed by treatment with ZK53, IKE, or their combination, along with a ferroptosis inhibitor treatment group (**Figure 7A**).

The results demonstrated that while treatment with either low-dose ZK53 or IKE alone did not significantly inhibit tumor growth, ZK53 markedly enhanced tumor sensitivity to SLC7A11 inhibition (**Figure 7B**). Specifically, combination therapy led to a substantial reduction in tumor volume and weight, with the tumor size being less than half of that observed with single-agent treatments (**Figures 7C, D**). Further analysis of ferroptosis markers, including MDA and 4-HNE, showed significantly increased levels in the combination treatment group compared to the single-agent groups, indicating enhanced ferroptosis. Meanwhile, Ki-67 levels were notably decreased in the combination group, reflecting reduced tumor cell proliferation (**Figures 7E, G–I**). This synergistic effect was effectively reversed by the ferroptosis inhibitor Lip-1, further confirming the role of ZK53 in promoting ferroptosis. Notably, no significant changes in body weight were observed during the treatment (**Figures 7F**), indicating that the combination therapy did not cause overt toxicity or adverse effects in the animals.

In conclusion, ZK53 enhances tumor regression by amplifying ferroptosis, highlighting its potential as a powerful ferroptosis-sensitizing agent for cancer therapy.

## Discussion

Previous studies have demonstrated that the ClpP agonist ONC201 promotes ferroptosis through GPX4 inhibition (54). However, ONC201 is a polypharmacological agent that targets multiple pathways (55). Specifically, it activates mitochondrial ClpP, induces TRAIL, and antagonizes dopamine receptors D2/D3, which complicates the precise determination of ClpP’s contribution to ONC201-mediated ferroptosis. In contrast, ZK53 is a novel ClpP agonist characterized by a distinct chemical structure compared to existing ClpP activators, and it exhibits species specificity (40). This specificity positions ZK53 as a more suitable candidate for assessing the potential of ClpP as a targeted therapeutic strategy in cancer, thereby minimizing the off-target effects and inherent unpredictability associated with polypharmacological agents. The findings of this study provide robust evidence that the ferroptosis-sensitizing effect of ZK53 is ClpP-dependent, with mitochondrial dysfunction induced by ZK53 playing a critical role in enhancing ferroptosis sensitivity.

Interestingly, although the ferroptosis-sensitizing effect of ZK53 is significantly diminished in ClpP knockdown cells, a residual effect remains. We hypothesize that this residual effect may arise from two potential mechanisms. First, incomplete degradation of ClpP by RNA interference (RNAi) may allow for residual ClpP activity, which could partially mediate the ferroptosis-promoting effect of ZK53. Second, ZK53 may target ferroptosis-related pathways beyond ClpP, as previous studies have suggested that ZK53 can activate the DNA damage response (DDR) signaling pathway through an unknown mechanism, leading to cell cycle arrest (40), which has been previously associated with ferroptosis (56). Whether this residual effect is due to experimental limitations or reflects the existence of an unknown mechanism remains to be determined, and further experimental investigation is required to fully elucidate these possibilities.

ZK53 activates HsClpP, resulting in the degradation of mitochondrial electron transport chain (ETC) subunits and disruption of oxidative phosphorylation (OXPHOS) (40). The ETC plays a crucial role in ferroptosis through the generation of reactive oxygen species (ROS) and coenzyme Q within mitochondria (30, 31, 57). As a key regulator of ferroptosis, targeting the ETC has emerged as a promising therapeutic strategy for ferroptosis-associated cancers. However, a recent study reported neurological toxicity in patients treated with the ETC complex I inhibitor IACS-010759, raising concerns about the safety of ETC-targeting therapies. Although ZK53 has not shown significant toxicity in mouse models, further comprehensive investigations into its safety profile are essential to fully assess its therapeutic potential.

## Conclusion

**Figure 8:**
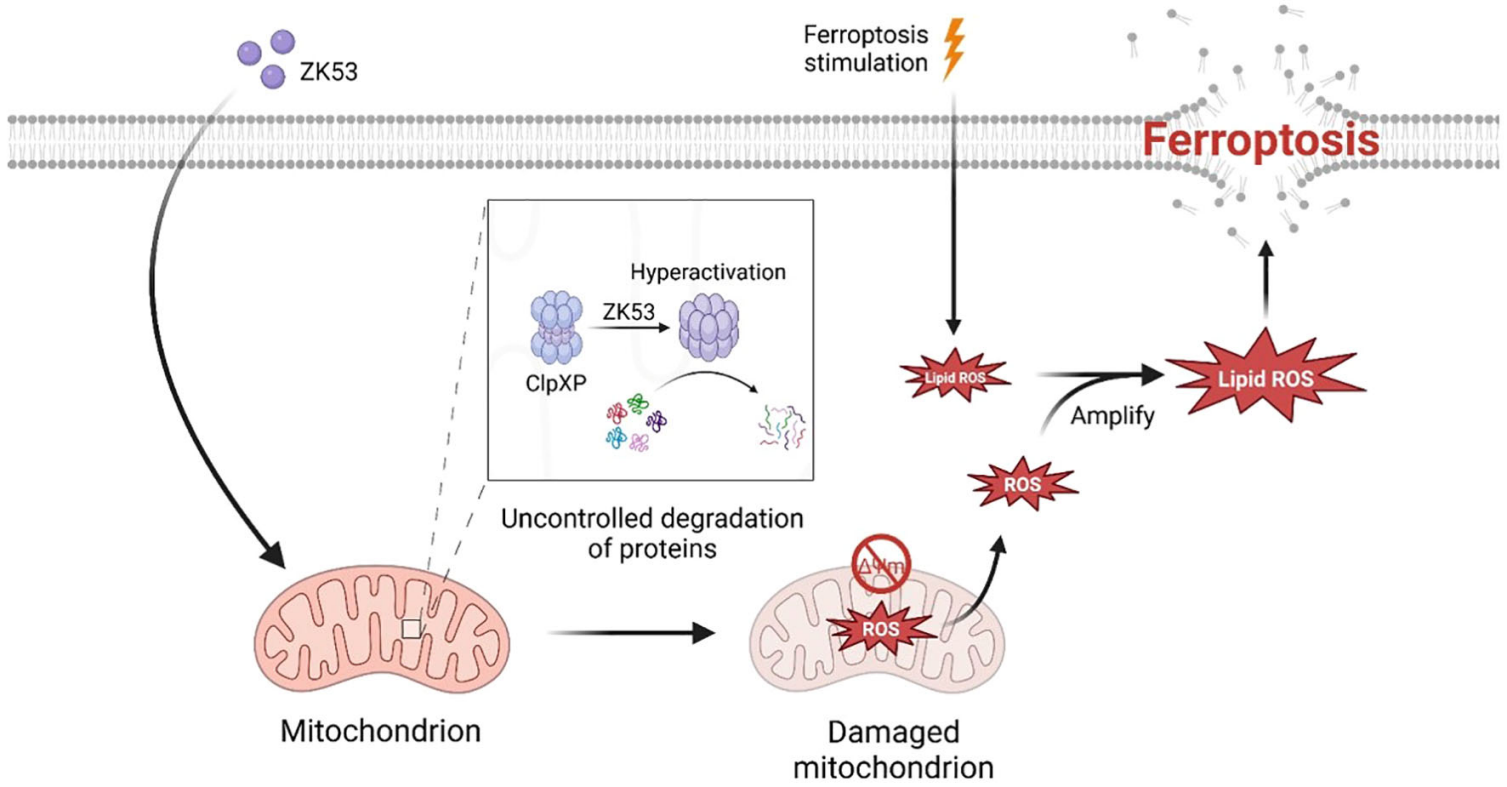
Proposed model for ZK53-induced hyperactivation of ClpP to amplify lipid peroxidation and promote ferroptosis. Treatment with ZK53 leads to the hyperactivation of mitochondrial ClpP, resulting in the uncontrolled degradation of mitochondrial proteins. This disruption causes mitochondrial dysfunction and the generation of mitochondrial ROS. These ROS further amplify lipid peroxidation induced by ferroptosis inducers, thereby accelerating the progression of ferroptosis.

In this study, we identify ZK53 as a potent ferroptosis sensitizer in cancer cells (**Figures 8**). High-throughput screening and functional validation reveal that ZK53 enhances sensitivity to ferroptosis inducers, such as RSL3 and erastin, via a ClpP-dependent mechanism. Mechanistically, ZK53 promotes ferroptosis via mitochondrial dysfunction, and this pro-ferroptotic effect can be reversed by mitochondrial ROS scavengers. *In vivo*, ZK53 enhances tumor sensitivity to ferroptosis inducers, significantly inhibiting tumor growth without causing overt toxicity.

Together, these findings position ZK53 as a promising therapeutic agent that sensitizes cancer cells to ferroptosis through ClpP activation and mitochondrial dysfunction. Further studies are needed to fully understand its mechanisms and assess its clinical safety and efficacy.

## Data Availability

The original contributions presented in the study are included in the article/supplementary material. Further inquiries can be directed to the corresponding author.
